# Emerging, Non-PCV13 Serotypes 11A and 35B of *Streptococcus pneumoniae* Show High Potential for Biofilm Formation *In Vitro*

**DOI:** 10.1371/journal.pone.0125636

**Published:** 2015-04-30

**Authors:** Mirian Domenech, Diana Damián, Carmen Ardanuy, Josefina Liñares, Asunción Fenoll, Ernesto García

**Affiliations:** 1 Centro de Investigaciones Biológicas, CSIC, Madrid, Spain; 2 CIBER de Enfermedades Respiratorias (CIBERES), Madrid, Spain; 3 Hospital Universitari de Bellvitge–Universitat de Barcelona–Fundació Privada Institut d’Investigació Biomèdica de Bellvitge, Barcelona, Spain; 4 Centro Nacional de Microbiología, Instituto de Salud Carlos III, Madrid, Spain; Faculdade de Medicina de Lisboa, PORTUGAL

## Abstract

**Background:**

Since the use of pneumococcal conjugate vaccines PCV7 and PCV13 in children became widespread, invasive pneumococcal disease (IPD) has dramatically decreased. Nevertheless, there has been a rise in incidence of *Streptococcus pneumoniae* non-vaccine serotypes (NVT) colonising the human nasopharynx. Nasopharyngeal colonisation, an essential step in the development of *S*. *pneumoniae*-induced IPD, is associated with biofilm formation. Although the capsule is the main pneumococcal virulence factor, the formation of pneumococcal biofilms might, in fact, be limited by the presence of capsular polysaccharide (CPS).

**Methodology/Principal Findings:**

We used clinical isolates of 16 emerging, non-PCV13 serotypes as well as isogenic transformants of the same serotypes. The biofilm formation capacity of isogenic transformants expressing CPSs from NVT was evaluated *in vitro* to ascertain whether this trait can be used to predict the emergence of NVT. Fourteen out of 16 NVT analysed were not good biofilm formers, presumably because of the presence of CPS. In contrast, serotypes 11A and 35B formed ≥45% of the biofilm produced by the non-encapsulated M11 strain.

**Conclusions/Significance:**

This study suggest that emerging, NVT serotypes 11A and 35B deserve a close surveillance.

## Introduction

*Streptococcus pneumoniae* (pneumococcus) is a leading human pathogen that naturally inhabits the upper respiratory tract. It usually colonises the associated mucosal surfaces in early childhood, and persists as a symptomless commensal (carrier state) in the nasopharynx [1]. Carriage is higher in children less than 5 years and decreases with age and is generally higher in developing countries and among economically deprived populations. Once carriage is established, however, *S*. *pneumoniae* may invade several sterile sites, leading to what is known as invasive pneumococcal disease (IPD). Indeed, the pneumococcus is responsible for episodes of bacteraemic community-acquired pneumonia, bacteraemia and meningitis, mainly in children, the elderly, and immunocompromised patients [2]. In addition, pneumococci are the main ethiologic agent of non-bacteraemic community-acquired pneumonia and a major cause of other non-invasive diseases such as acute otitis media, sinusitis, and conjunctivitis.

The pneumococcal capsule is composed of polysaccharides (capsular polysaccharide or CPS) that, in most cases, are covalently bound to the cell wall. At least 95 different pneumococcal serotypes are currently known, each with a biochemically different CPS. The capsule is the main pneumococcal virulence factor since it allows the bacterium to evade the host immune system by blocking phagocytosis [2]. Pneumococcal vaccines [3] are based on combinations of CPSs, and include a) a 23-valent pneumococcal polysaccharide vaccine; b) a 7-valent conjugate vaccine (PCV7), directed at serotypes 4, 6B, 9V, 14, 18C, 19F and 23F, but no longer available in the market; c) a 10-valent conjugate vaccine (PCV10), licensed in Europe (but not in the United States) and directed at serotypes 1, 5, and 7F in addition to the serotypes included in PCV7; and d) a 13-valent pneumococcal conjugate vaccine (PCV13) that was licensed five years ago; this subsequently replaced PCV7. PCV13 includes six conjugate CPSs of serotypes 1, 3, 5, 6A, 7F, and 19A in addition to the serotypes included in PCV7.

The widespread use of conjugate vaccines has been very effective in reducing cases of IPD, although increases in disease caused by non-vaccine serotypes (NVT) (“serotype replacement”) have subsequently offset some of these reductions. Although the PCV7 serotype 6B appears to offer cross-protection against the non-PCV7 serotype 6A, this is not the case for 19F and the non-PCV7 serotype 19A [4]. Indeed, the incidence of infections caused by 19A multiresistant pneumococcus has increased since PCV7 vaccination became common. NVT are also on the rise in the current post-PCV7/PCV13 era. Surveillance program results suggest that pneumococci of various serogroups/serotypes not included in PCV13 (*e*.*g*., 11, 12, 15, 22F, 23A, 23B, 33F, 24, 34, and 35B) are rapidly increasing in prevalence worldwide [5–8]. Serotype 6C pneumococci, which are good biofilm formers [9] and whose CPS is not included in PCV13, were not investigated further.

Concerns about serotype replacement have influenced recent policy discussions [10]. Predicting the amount of future replacement is difficult since the reasons underlying a particular NVT increases after vaccine introduction are not fully understood. In the years immediately preceding PCV13 introduction, no single non-PCV13 serotype seemed an obvious candidate for replacement, based on current rates of disease, antimicrobial resistance, and carriage data. We recently proposed that quantifying the *in vitro* biofilm formation capacity of isogenic *S*. *pneumoniae* transformants expressing different CPSs might help predict the emergence (and eventual expansion) of NVT that are prone to colonise the human nasopharynx [9]. In that work we showed that clinical isolates and isogenic pneumococcal transformants of serotypes 19F and 19A (but not those of serotypes 19B and 19C) are capable of forming substantial amounts of biofilm *in vitro*. Strains of serogroup 6 also showed significant biofilm-forming capacity.

In the present study, 16 isogenic pneumococcal transformants expressing CPSs of non-PCV13 serotypes were constructed to determine whether biofilm formation can reliably predict the emergence of NVT of *S*. *pneumoniae* that colonise the human nasopharynx.

## Materials and Methods

### Media, growth conditions, DNA purification, and genetic transformation

Table 1 shows the pneumococcal strains examined. Clinical isolates of *S*. *pneumoniae* were from the collection of the Spanish Pneumococcal Reference Laboratory (SPRL; Centro Nacional de Microbiología ISCIII, Majadahonda, Madrid, Spain). All strains were grown in liquid CpH8 medium supplemented (or not) with 0.08% yeast extract (C+Y medium), or in solid medium involving trypticase soy agar or D agar supplemented with 5% defibrinated sheep blood (Thermo Scientific; Hampshire, England) [11]. Growth was controlled by measuring the optical density at 550 nm (OD_550_).

**Table 1:** Strains of *S*. *pneumoniae* used in this study^a^.

Strain	Description/serotype (source)	Reference/Source^b^
M11	Non-encapsulated strain derived from R6 (Hex^–^, *lytA* ^+^)	[9]
2977/13	8 (blood)	SPRL
3013/13	8 (blood)	SPRL
6028/95	8 (blood)	[12]
P012	M11 transformant with DNA from strain 6028/95; serotype 8	D. Llull, CIB
2965/13	9N (bronchial aspirate)	SPRL
3015/13	9N (blood)	SPRL
P238	M11 transformant with DNA from strain 3015/13; serotype 9N	This study
2960/13	10A (blood)	SPRL
3011/13	10A (blood)	SPRL
P245	M11 transformant with DNA from strain 3011/13; serotype 10A	This study
2901/13	11A (sputum)	SPRL
2963/13	11A (blood)	SPRL
P242	M11 transformant with DNA from strain 2963/13; serotype 11A	This study
2951/13	12F (blood)	SPRL
2995/13	12F (blood)	SPRL
SSISP12F/1	12F (not determined)	Staten Seruminstitut
P020	M11 transformant with DNA from strain SSISP12F/1; serotype 12F	D. Llull, CIB
2949/13	15A (blood)	SPRL
2957/13	15A (blood)	SPRL
P247	M11 transformant with DNA from strain 2949/13; serotype 15A	This study
2948/13^c^	15B/C (bronchial aspirate)	SPRL
2990/13^c^	15B/C (otic exudate)	SPRL
SSISP15B/1	15B (sputum)	Staten Seruminstitut
P013	M11 transformant with DNA from strain SSISP15B/1; serotype 15B	D. Llull, CIB
1572/14	16F (blood)	SPRL
1607/14	16F (blood)	SPRL
P246	M11 transformant with DNA from strain 1572/14; serotype 16F	This study
3014/13	22F (blood)	SPRL
3022/13	22F (blood)	SPRL
P244	M11 transformant with DNA from strain 3014/13; serotype 22F	This study
1544/14	23A (cerebrospinal fluid)	SPRL
3004/13	23A (blood)	SPRL
P240	M11 transformant with DNA from strain 3004/13; serotype 23A	This study
2971/13	23B (sputum)	SPRL
3000/13	23B (aqueous humor)	SPRL
P248	M11 transformant with DNA from strain 3000/13; serotype 23B	This study
2966/13	24F (blood)	SPRL
3017/13	24F (blood)	SPRL
P224	M11 transformant with DNA from strain 3017/13; serotype 24F	[9]
436/96	31 (not determined)	SPRL
2847/13	31 (sputum)	SPRL
2959/13	31 (blood)	SPRL
P016	M11 transformant with DNA from strain 436/96; serotype 31	D. Llull, CIB
2975/13	33F (blood)	SPRL
2922/13	33F (blood)	SPRL
SSISP33F/1	33F (not determined)	Staten Seruminstitut
P017	M11 transformant with DNA from strain SSISP33F/1; serotype 33F	D. Llull, CIB
2967/13	35F (sputum)	SPRL
3012/13	35F (blood)	SPRL
P239	M11 transformant with DNA from strain 2967/13; serotype 35F	This study
2896/13	35B (blood)	SPRL
2943/13	35B (conjunctivitis)	SPRL
P241	M11 transformant with DNA from strain 2943/13; serotype 35B	This study

^**a**^Strains are ordered by serotype.

^**b**^SPRL, Spanish Pneumococcal Reference Laboratory (Centro Nacional de Microbiología ISCIII, Majadahonda, Madrid, Spain).

^**c**^Whether these strains are of serotype 15B or 15C could not be determined. While some studies consider 15B and 15C to be separate entities, their coding regions differ by only a single TA tandem repeat in the *wciZ* gene, which results in a single difference in the *O*-acetyl structure. The CPS of serotype 15B is the *O*-acetylated variant of that possessed by 15C CPS [13]. Although upon first isolation from a patient serotypes 15B and 15C can be accurately differentiated, after few *in vitro* passages, this is not usually possible since these serotypes can interconvert. Therefore, serotypes 15B and 15C were considered together in the present study.

*S*. *pneumoniae* chromosomal DNA was purified from clinical strains and encapsulated, isogenic transformants constructed by transformation of strain M11, as described elsewhere [9]. The selection of transformants was made by enriching those encapsulated by successive transfer to C+A medium (CpH8 containing 0.08% of bovine seroalbumin) supplemented with 0.5 μl/ml anti-R antiserum (which agglutinates only non-encapsulated pneumococci) [9]. Serotyping of *S*. *pneumoniae* was kindly performed by D. Vicioso at the SPRL.

### Biofilm formation assay, quantification, and statistical analysis

The optimal conditions for biofilm formation by pneumococcal cells on polystyrene microtitre plates have been previously described [11,14]. In short, pneumococcal cells were grown in C+Y medium at 37°C to an OD_550_ of 0.5–0.6 and diluted (1:100) in fresh C+Y medium. Aliquots (200 μl) were dispensed into each well in triplicate, and the plate incubated at 34°C for 6 h. Growth (OD_595_) was measured and the biofilm formed was stained with 1% crystal violet [11]. In the present study, a strain was considered as “good” or “intermediate” biofilm producer when it formed ≥45% or between 10% and 30% of the biofilm formed by the control, non-encapsulated M11 strain respectively. The data for biofilm formation include the mean ± standard error of at least three independent experiments, each performed in triplicate. Statistical significance was examined using the Student *t* test. For multiples comparisons, one-way analyses of variance (ANOVA) were performed, followed by Tukey’s *post hoc* test when the ANOVA rejected the null hypothesis. The SAS 9.3 statistical packge (SAS Institute, Cary, NC) was used for all analyses. Differences were considered statistically significant when *P* <0.05.

## Results

**Fig 1:**
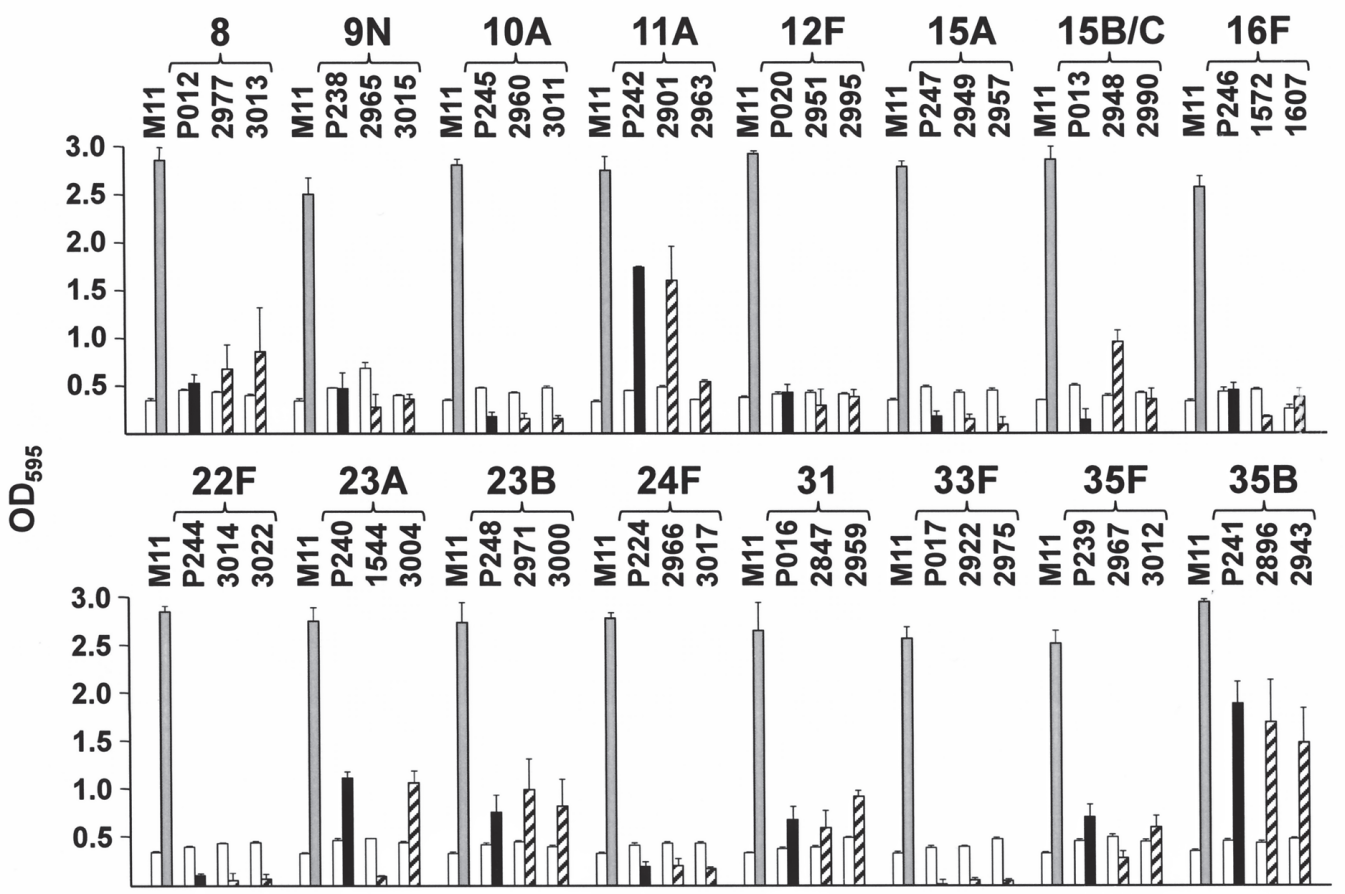
Biofilm formation. Growth and biofilm formation by *S*. *pneumoniae* clinical isolates of the indicated serotypes, and their encapsulated M11 transformants. In all cases, open bars indicate bacterial growth. Hatched and blackened bars indicate, respectively, biofilm formation by either clinical isolates or their encapsulated M11 transformants. Grey bars correspond to biofilm formation by the M11 strain. Biofilm formation by the M11 strain was determined in all experiments with mean values between serotypes that were not significantly different (*P* >0.05 using one-way ANOVA).

Fig 1 shows the biofilm formation capacity of clinical isolates of *S*. *pneumoniae* belonging to 16 non-PCV13 serotypes (Table 1). Two clinical isolates of each serotype were analysed in detail. Although pneumococci of every serotype tested produced less biofilm than the non-encapsulated strain M11, significant differences were noted in the biofilm formation capacity of several pairs of clinical strains of the same serotype: *e*.*g*., strains 2901 and 2963 (serotype 11A), 2948 and 2990 (serotype 15B/C), and 1544 and 3004 (serotype 23A). This confirms previous observations indicating that the genetic background, and not only the CPS, modulates pneumococcal attachment to the artificial substrate [9,11,15].

Since *in vitro* biofilm formation by pneumococcus depends on many different genes and/or biochemical traits, the use of isogenic transformants (*i*.*e*., strains differing only by a single trait) is necessary when the influence of CPS in biofilm formation is to be measured. To this end, the non-encapsulated M11 strain was transformed with DNA prepared from the corresponding clinical isolates, and at least two independent transformants of each serotype were analysed. Since initial experiments revealed no significant differences between them, only one transformed strain of each serotype was studied further. The serotype 35B strains were the best biofilm producers; the P241 transformant strain produced ≈50% of the biofilm formed by M11. In addition, the P242 transformant (serotype 11A) was capable of synthesizing up to 45% of the biofilm produced by its non-encapsulated progenitor. Intermediate biofilm formation was noted for the isogenic *S*. *pneumoniae* transformants of serotype 23A. In contrast, serotype 33F pneumococci (whether clinical isolates or M11 transformant) were unable to produce any substantial quantity of biofilm *in vitro* (Fig 1).

**Fig 2:**
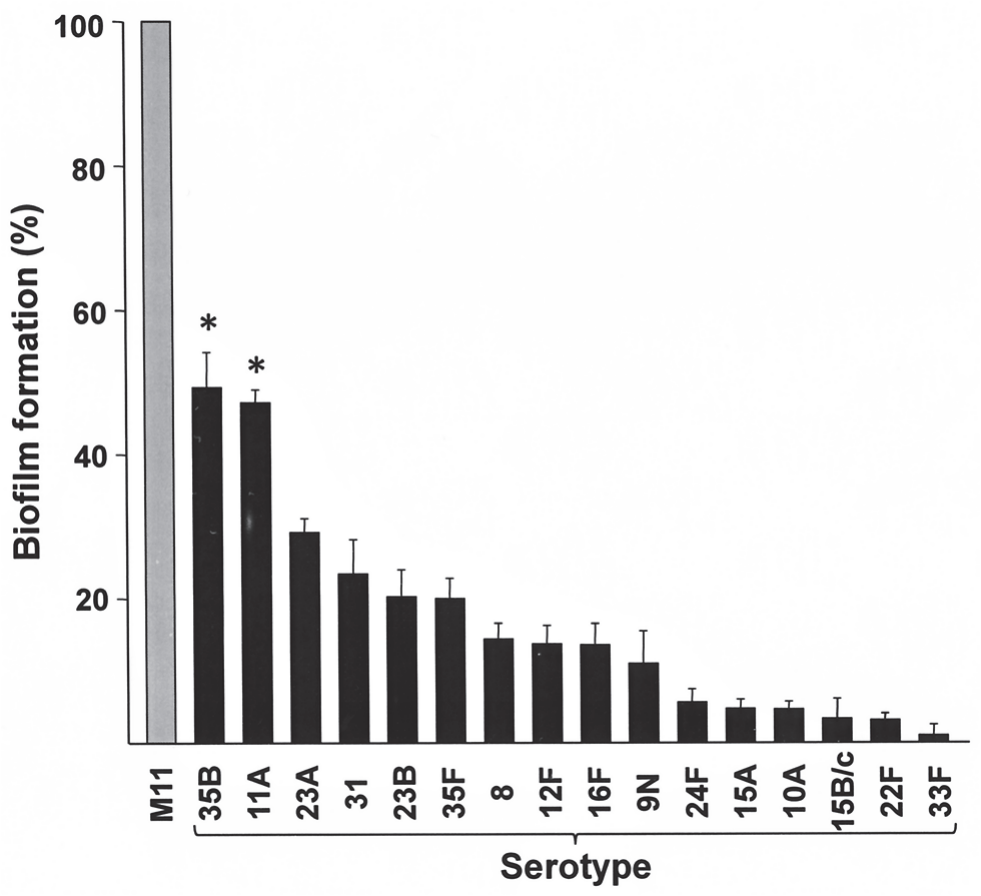
Relative biofilm formation. Relative biofilm formation capacity of M11 isogenic transformants with non-PCV13 serotypes compared to that of their parental non-encapsulated strain (M11). The percentages shown are the mean ± standard error of at least three independent experiments, each performed in triplicate. Relative biofilm formation was significantly different between serotypes (*, *P* <0.0001 in a one-way ANOVA). The Tukey’s *post hoc* test showed significant differences in biofilm formation between serotypes 35B and 11A, and the rest of serotypes. No significant differences were observed between the biofilm-forming capacity of serotypes 35B and 11A.

Some variation was noted in the growth rate and/or biofilm formation capacity of the different strains (Fig 1). Consequently, the biofilm formation values for the encapsulated strains were normalised for the OD of the culture measured as stated in Materials and Methods, and the presented percentages were calculated in relation to the parental strain M11 [11]. Fig 2 shows the relative biofilm formation capacity of the isogenic transformants of the non-PCV13 serotypes compared with that of M11. Every serotype analysed formed significantly less biofilm than the non-encapsulated strain, although serotypes 35B and 11A formed significantly more biofilm than the other serotypes analysed.

## Discussion

Recent years have seen continuous interest in the impact of pneumococcal conjugate vaccines on nasopharyngeal colonisation [16]. In fact, surveillance of colonisation has become an important component of the vaccination monitoring process in the post-licensure setting. Nasopharyngeal colonisation, which is associated with biofilm formation, is an essential step in the development of *S*. *pneumoniae* disease and prevention of colonisation may reduce host-to-host transmission [17,18]. Moreover, colonisation by multiple serotypes (co-colonisation), which appears to be much more prevalent than previously envisaged [19], is an important factor to consider as it facilitates horizontal gene transfer [20]. Thus, the ability of pneumococcal serotypes to form biofilms—which a previous publication [9] and the present study show can be rapidly tested *in vitro*—could be used to help predict the expansion of NVT. This method has recently shown isogenic transformants and clinical isolates of serotype 19A and serogroup 6, which were emerging during the PCV7 era, to be good biofilm-formers [9], and many studies have shown that the non-PCV13 serotypes analysed in the present study underwent a rapid increase in prevalence after PCV13 implementation. Nevertheless, no previous studies have assessed the ability of these serotypes to form biofilms *in vitro*.

The present results clearly show that most (14 out of 16) of the clinical isolates or isogenic transformants expressing the CPSs of NVT were unable to form substantial amounts of biofilm *in vitro*. Although an inverse relationship between CPS and biofilm formation exists [14], it has previously been shown that a minimum amount of CPS is necessary for efficient nasopharyngeal colonisation in mice [15]. The situation, however, is far from clear. For example, *S*. *pneumoniae* isolates of serotypes 8, 22F and 33F, which were found both in carriage and causing IPD, and which are potentially highly invasive [21], failed to produce substantial amounts of biofilms *in vitro* in the present study. This finding strongly suggests that *in vitro* biofilm formation is mostly unrelated to IPD-inducing capacity. It should be underlined that the CPSs of serotypes 22F and 33F are included in a 15-valent pneumococcal conjugate vaccine that is currently under pre-clinical evaluation [22].

The present results also show that the biofilm formation capacity of the corresponding isogenic transformants almost paralleled (and in some cases exceeded) that of the corresponding clinical isolates. One exception was seen, however: the clinical isolate 2948 (serotype 15B/C) (Fig 1). This strain showed significantly more biofilm formation capacity than strain P013, an M11 transformant of serotype 15B obtained using DNA from the reference strain SSISP15B/1 (Table 1). It may be that strain 2948 has a genetic background more prone to allow biofilm formation than strains M11 and 2990. It should be reminded that the serotypes of strains 2948 and 2990 were not determined; they might not, therefore, belong to the same serotype (Table 1, see footnote c).

In sharp contrast to the other serotypes tested here, isogenic transformants expressing the CPS of serotype 35B or 11A were capable of producing substantial amounts of biofilm *in vitro*, forming ≥45% of the amount of biofilm produced by the non-encapsulated M11 strain (Fig 2). This may explain the current prevalence of these serotypes in the human nasopharynx [23,24].

**Fig 3:**
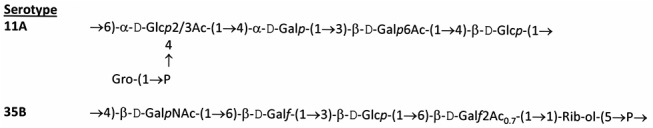
Primary structures of the capsular polysaccharides of *S*. *pneumoniae* serotypes 11A and 35B. Data were taken from previous studies [13,27].

Many factors appear to influence the colonisation capacity of pneumococcal serotypes. For example, a previous study showed CPS (the major determinant of surface charge in *S*. *pneumoniae*) to be associated with colonisation capacity [25]. With the notable exceptions of serotypes 19A and 11A, higher net negative surface charge was associated with higher resistance to nonopsonic, neutrophil-mediated killing as well as higher carriage prevalence. It has also been suggested that a direct relationship exists between the success (in terms of relative numbers) of a serotype during carriage and the biochemistry of its CPS [26]. In fact, it has been recently shown that the CPSs of clinical isolates and isogenic transformants expressing the CPS of serotypes 19F and 19A, and all members of serogroup 6 (which are good biofilm producers), all include the disaccharides α-D-Glc*p*-(1→2)-α-L-Rha*p*-(1→ and α-D-Glc*p*-(1→3)-α-L-Rha*p*-(1→ [9]. However, neither of these disaccharides is present in the capsules of serotypes 11A or 35B (Fig 3). In addition, no chemical similarities between serotypes 11A and 35B CPSs are obvious—although both contain *O*-acetylated galactose residues. Recently, Calix *et al*. [27] showed that the newly discovered, invasive serotype 11E contains loss-of-function mutations in the capsule *O*-acetyltransferase gene *wcjE*, and does not express β-D-Gal*p*6Ac at all. It is remarkably that, although frequently found in carriers, serotype 11A shows low invasiveness due to ficolin-2 recognition of *O*-acetylated capsule epitopes and the consequent activation of the lectin complement pathway activation [28]. It it well known that some serotypes are rarely found in carriage although they are known to cause disease, *e*.*g*., serotypes/serogroups 1, 3, 5, and 7 [29], but the reasons underlying how and when pneumococcal colonisation will result in dissemination and disease remain poorly understood [16,30–32].

There is increasing interest in the interactions between the different species of the nasopharyngeal microbiota, because of the possibility that PCV vaccination might affect the ecology of the nasopharynx, which could have clinical consequences. This topic is subjected to debate; for example, an inverse relation between carriage of pneumococcus and *Staphylococcus aureus* has been found in some studies [33,34], but not in others [35–37].

Evaluating vaccine efficacy for protection against colonisation with *S*. *pneumoniae* and other bacterial pathogens is an area of growing interest [38]. Conjugate vaccines lead to a reduction in the carriage prevalence of vaccine-serotype pneumococci in both vaccinated and unvaccinated individuals and a reduction in hospital admissions for invasive and non-invasive pneumococcal pneumonia in children younger than 5 years, as well as in some adult age groups, indicating herd protection [39]. Interestingly, it has been shown that estimating the adult burden of pneumococcal disease from bacteraemic pneumococcal pneumonia data alone significantly underestimates the true burden of disease in adults. For every case of bacteraemic pneumococcal pneumonia, it has been estimated that there are at least 3 additional cases of non-bacteraemic pneumococcal pneumonia [40]. Management of these infections is potentially being compromised by the increasing resistance of the pathogen to antibiotics commonly used to treat these infections [41]. Of note, increasing antibiotic resistance has been recently detected both in serotype 35B [42,43] and serotype 11A *S*. *pneumoniae* isolates [44]. However, since pneumococcal vaccines have been dessigned for the active prevention of IPD and pneumococci of serotypes 11A and 35B only seldom cause IPD [45–47], it is questionable whether these serotypes should be included in future higher-valency PCVs. Yet, further studies of surveillance are needed in order to detect the emergence of non PCV-13 serotypes and allow rational vaccine design, implementation and continued effective control of pneumococcal disease.
